# Impact of dispatcher-assisted cardiopulmonary resuscitation on neurologically intact survival in out-of-hospital cardiac arrest: a systematic review

**DOI:** 10.1186/s13049-021-00875-5

**Published:** 2021-05-24

**Authors:** Kristine Elisabeth Eberhard, Gitte Linderoth, Mads Christian Tofte Gregers, Freddy Lippert, Fredrik Folke

**Affiliations:** 1 Copenhagen Emergency Medical Services, Copenhagen, Denmark; 2 Department of Clinical Medicine, University of Copenhagen, Copenhagen, Denmark; 3 Department of Anesthesia and Intensive Care, Copenhagen University Hopsital – Bispebjerg and Frederiksberg, Copenhagen, Denmark; 4 Department of Cardiology, Copenhagen University Hospital – Herlev and Gentofte, Copenhagen, Denmark

**Keywords:** Dispatcher-assisted CPR, Telephone-assisted CPR, T-CPR, DA-CPR, Cardiopulmonary resuscitation, Out-of-hospital cardiac arrest, Cardiac arrest, Emergency medical services, Medical dispatch, Systematic review

## Abstract

**Background:**

Dispatcher-assisted cardiopulmonary resuscitation (DA-CPR) increases neurologically intact survival in out-of-hospital cardiac arrest (OHCA) according to several studies. This systematic review summarizes neurologically intact survival outcomes of DA-CPR in comparison with bystander-initiated CPR and no bystander CPR in OHCA.

**Methods:**

The systematic review was conducted according to the PRISMA guidelines. All studies including adult and/or pediatric OHCAs that compared DA-CPR with bystander-initiated CPR or no bystander CPR were included. Primary outcome was neurologically intact survival at discharge, one-month or longer. Studies were searched for in PubMed (MEDLINE), EMBASE, and the Cochrane Library databases. The risk of bias was evaluated using the Newcastle-Ottawa Scale.

**Results:**

The search string generated 4742 citations of which 33 studies were eligible for inclusion. Due to overlapping study populations, the review included 14 studies. All studies were observational. The study populations were heterogeneous and included adult, pediatric and mixed populations. Some studies reported only witnessed cardiac arrests, arrests of cardiac ethiology, and/or shockable rhythm. The individual studies scored between six and nine on the Newcastle-Ottawa Scale of risk of bias. The median neurologically intact survival at hospital discharge with DA-CPR was 7.0% (interquartile range (IQR): 5.1–10.8%), with bystander-initiated CPR 7.5% (IQR: 6.6–10.2%), and with no bystander CPR 4.4% (IQR: 2.0–9.0%) (four studies). At one-month neurologically intact survival with DA-CPR was 3.1% (IQR: 1.6–3.4%), with bystander-initiated CPR 5.7% (IQR: 5.0–6.0%), and with no bystander CPR 2.5% (IQR: 2.1–2.6%) (three studies).

**Conclusion:**

Both DA-CPR and bystander-initiated CPR increase neurologically intact survival compared with no bystander CPR. However, DA-CPR demonstrates inferior outcomes compared with bystander-initiated CPR. Early CPR is crucial, thus in cases where bystanders have not initiated CPR, DA-CPR provides an opportunity to improve neurologically intact survival following OHCA. Variability in OHCA outcomes across studies and multiple confounding factors were identified.

**Supplementary Information:**

The online version contains supplementary material available at 10.1186/s13049-021-00875-5.

## Background

Cardiac arrest is one of the leading causes of death worldwide [1–3], and despite countless initiatives, the overall survival remains below 12% [2–4]. Each minute defibrillation is delayed and cardiopulmonary resuscitation (CPR) is not provided, the probability of survival is reduced by 7–10% [5]. If immediate CPR is provided until defibrillation, the probability of survival only declines by 3–4% per minute [5, 6]. The effect of bystander CPR can potentially double the survival rates for witnessed OHCAs, and more importantly, double the rate of neurologically intact survival [7, 8].

Several studies have demonstrated that community CPR training can increase bystander CPR rates but the majority of training programs do not reach where they have the biggest effect: At home amongst the elderly population [9–13]. Furthermore it is well known that retention of CPR skills is poor without regular training, and only few bystanders attend regular training [10, 11, 14–16].

To strengthen the chain of survival, the concept *dispatcher-assisted CPR* (DA-CPR) was developed and it is now recommended by the Global Resuscitation Alliance, the Resuscitation Academy, and in the guidelines of both the American Heart Association and the European Resuscitation Council [17–23]. In recent years numerous studies from around the world have been published on the effect of DA-CPR: It has the potential to increase bystander CPR rates to more than 50% for witnessed arrests, and diminishes time to first compression to less than 3 min – two factors inevitably associated with a higher probability of survival including neurologically intact survival [7, 8, 24–26]. Simulation studies have investigated the quality of DA-CPR, and though the quality rarely meets the requirements of guidelines, it may be comparable with CPR provided by a trained bystander [27, 28].

This systematic review aims to assess the effects of DA-CPR on neurologically intact survival in patients with OHCA, as well as survival to discharge, one-month survival or longer, and the return of spontaneous circulation (ROSC).

## Methods

### Eligibility criteria and outcome parameters

This systematic review was conducted according to the PRISMA (Preferred Reporting Items for Systematic Reviews and Meta-Analyses) guidelines by three reviewers (KEE, GL, MTG) [29, 30]. The research question was developed on the PICOS framework recommended by the Cochrane Handbook [30, 31].

All studies with adult and/or pediatric OHCAs not witnessed by the Emergency Medical Services (EMS) that compared DA-CPR with bystander-initiated CPR and/or no bystander CPR before EMS arrival were included. DA-CPR was defined as OHCAs where bystanders provided CPR after instructions from the dispatcher. Bystander-initiated CPR was defined as OHCAs where bystanders initiated and provided CPR without assistance from the dispatcher. Studies only including traumatic OHCAs were excluded.

The primary outcome was neurologically intact survival at hospital discharge or at least one-month after OHCA. Neurologically intact survival was defined as Cerebral Performance Category (CPC) 1 or 2 or modified Rankin Scale (mRS) 0–3. CPC level 1 represents “good recovery”, and CPC 2 means “moderate disability / disabled but independent” [32, 33].

Secondary outcomes were survival to hospital discharge, or at least one-month survival, and prehospital ROSC or ROSC at hospital arrival.

Only studies written in English were eligible. Simulation studies, case reports, and conference abstracts were excluded. No restrictions on publication and study years were applied.

### Information sources and search

The following databases were systematically searched: PubMed (MEDLINE), EMBASE, and the Cochrane Library. In collaboration with a professional librarian from Copenhagen University Library, the search was designed to include all studies covering both cardiopulmonary resuscitation and dispatcher assistance. Table 1 provides the search string designed for the PubMed (MEDLINE) database. The search was modified to fit EMBASE and the Cochrane Library databases. The search was performed on the April 14th 2020 in all three databases.

**Table 1:** Final search string entered to the PubMed database^a^

**1**	(((((((((((((((dispatch) OR dispatcher) OR dispatchers) OR dispatched) OR telephone) OR telephone[MeSH Terms]) OR “emergency medical services”) OR emergency medical services[MeSH Terms]) OR “emergency medical service”) OR emergency medical dispatch[MeSH Terms])) **AND** ((((((((((“assistance”) OR “assisted”) OR “assist”) OR “instruction”) OR “instructions”) OR “instructed”) OR “instruct”) OR “guidance”) OR “guided”) OR “guide”))) **OR** (((((((((((((((((dispatch-assistance) OR dispatcher-assistance) OR dispatch-assisted) OR dispatcher-assisted) OR dispatch-instruction) OR dispatcher-instruction) OR dispatch-instructions) OR dispatcher-instructions) OR dispatch-instructed) OR dispatcher-instructed) OR dispatcher-guidance) OR dispatch-guided) OR dispatcher-guided) OR DA-CPR) OR T-CPR) OR telemedicine[MeSH Terms]) OR remote consultation[MeSH Terms])))
**AND**	
**2**	((((((((((((((((((((resuscitation[MeSH Terms]) OR cardiopulmonary resuscitation[MeSH Terms]) OR ((cardiopulmonary) AND resuscitation)) OR cardiopulmonary resuscitation) OR (((cardio) AND pulmonary) AND resuscitation)) OR cardio pulmonary resuscitation) OR CPR) OR basic life support) OR (((basic) AND life) AND support)) OR basic cardiac life support) OR ((((basic) AND cardiac) AND life) AND support)) OR BLS) OR mouth-to-mouth resuscitation) OR compression) OR compressions) OR cardiac massage) OR cardiac massages) OR heart massage) OR heart massage[MeSH Terms]) OR heart massages)

^a^The search string was modified to fit EMBASE and the Cochrane Library databases

### Study selection

First, two independent reviewers screened all titles and abstracts for eligibility (KEE and GL/MTG). If any disagreement the full-text article was screened. The same reviewers independently screened the selected full-text articles. Any disagreement was resolved by discussion until consensus. Finally, the reference lists of all eligible studies were screened by the first reviewer (KEE) for studies that fulfilled the inclusion criteria [34].

Publications with overlapping study populations were considered duplicates, and only one was included in the final review. The study that reported the primary outcome, neurologically intact survival, was selected for inclusion. If equally relevant, the decision was based on whether secondary outcomes were reported, and finally, the study analyzing the effect of DA-CPR on the largest population was selected.

Outcomes were also assessed for the subgroup of studies including only witnessed arrests. Among the list of eligible studies according to PICOS, studies with witnessed arrests only were extracted, subsequently the criteria for removing publications with overlapping study populations were applied.

### Data collection process and data items

Data were extracted into predefined tables by the first reviewer (KEE) and confirmed by a second reviewer (GL/MTG). Data on study characteristics included: study design, inclusion and exclusion criteria, period, country of origin, data sources, and the existence of a standardized CPR instruction protocol and type of basic life support (BLS) instructed (e.g. compression only). Patient data included: age and sex of victim, witnessed status, etiology of arrest, the initial cardiac rhythm, and location of arrest, along with the total number of OHCA analyzed with/without DA-CPR. Finally, outcome measures extracted included: neurologically intact survival and survival at discharge and one-month or longer, and ROSC.

### Risk of bias in individual studies

Risk of bias was assessed using the Newcastle-Ottawa Scale by two reviewers (KEE and GL/MTG) [35]. The Newcastle-Ottawa Scale is developed to assess the quality of non-randomized studies by rating three domains of case-control and cohort studies: 1) Selection of study groups, 2) comparability of groups, and 3) ascertainment of exposure or outcome. A study can be awarded from zero to nine stars; nine stars representing a low risk of bias. In the *comparability domain*, this review defined the most important factor as “witnessed arrest”, according to the findings by Sasson et al. 2010 [36]. A study was awarded one star for *comparability* if it controlled for “witnessed arrest”, either by study design in the inclusion/exclusion criteria, or in the analyses e.g. by conducting a logistic regression. An additional star was awarded if the study controlled for an additional factor. If less than 10% of the OHCAs were excluded from the analyses because of missing data, one star was awarded for *adequacy of follow-up* in the *outcome domain*.

### Summary measures

Median and interquartile range (IQR) for the primary and secondary outcome measures were calculated. For neurologically intact survival, unadjusted odds ratios and confidence intervals comparing DA-CPR and bystander-initiated CPR with no bystander were determined.

## Results

**Fig. 1:**
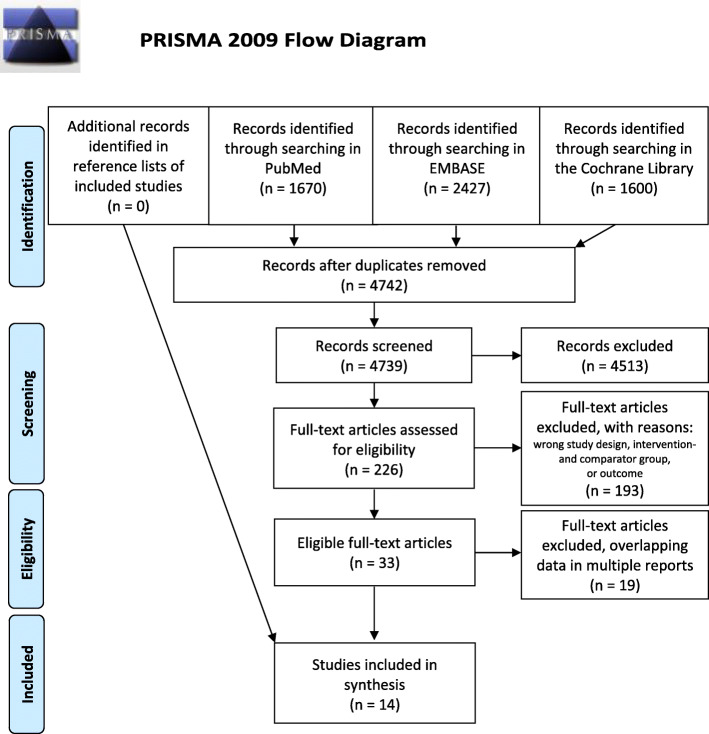
The PRISMA (Preferred Reporting Items for Systematic Reviews and Meta-Analyses) flow diagram of the study selection process

In total, 33 articles were identified and eligible for inclusion, of which 19 were excluded because of overlapping study populations (Supplementary Material Table 1). Finally, 14 articles were included [7, 17, 25, 37–47]. The study selection is illustrated in the standardized PRISMA flow diagram (Fig. 1) [48].

The 14 included studies are described in Table 2. They were conducted in eight different countries and three continents between 1981 and 2016; nine of 14 studies were conducted after 2010.

**Table 2:** Study Characteristics of Included Studies

Author, year of publication	Study period	Location	Study design	Data source / name of registry	Population/ Inclusion criteria	Dispatcher protocol	Quality, NOS score
Chang et al., 2018 [37]	01.2012–12.2015	Korea, nationwide	Retrospective cohort study	Korean National OHCA Registry, EMS run sheets for ambulance information & EMS cardiac arrest and dispatcher CPR registries.	Paediatric (≤18yo). Excluded: If no EMS resuscitation effort; if missing information on bystander CPR or neurological outcome.	Pediatric BLS, including ventilations < 9 years of age.	7 (Selection 4; Comparability 0; Outcome 3)
Eisenberg et al., 1985 [17]	05.1981–12.1982	USA, Washington, King County	Before-after study	Database of King County Emergency Services Division, medical records & telephone recordings.	Non-specified age; cardiac etiology.	Standard BLS starting with ventilations.	8 (Selection 4; Comparability 1; Outcome 3)
Goto et al., 2014 [38]	01.2008–12.2012	Japan, nationwide	Retrospective cohort study	Fire and Disaster Management Agency’s (FDMA) nationwide registry.	Paediatric (< 18 yo).	Compression only. Standard BLS to trained bystander.	7 (Selection 4; Comparability 0; Outcome 3)
Harjanto et al., 2016 [39]	04.2010–02.2013	Singapore	Before-after study	PAROS (Pan Asian Resuscitation Outcomes Study), telephone recordings, EMS- and patient records.	Non-specified age; cardiac etiology. Excluded: If a DNR order existed.	Compression only. Standard BLS if suspected hypoxia.	8 (Selection 4; Comparability 1; Outcome 3)
Hasselqvist et al., 2015 [7]	01.1990–12.2011	Sweden, nationwide	Retrospective cohort study	Swedish Cardiac Arrest Registry.	Non-specified age; witnessed arrest.	Instructions changed during study period.	8 (Selection 4; Comparability 1; Outcome 3)
Hiltunen et al., 2015 [40]	03.2010–08.2010	Finland, southern and eastern regions	Prospective cohort study	EMS registry with dispatch and EMS reports, The Finnish Population Information System & National Institute of Health and Welfare.	Non-specified age; witnessed arrest; shockable rhythm; arrest recognized by the dispatcher.	Compression only. Standard BLS if suspected hypoxia.	9 (Selection 4; Comparability 2; Outcome 3)
Kuisma et al., 2005 [41]	01.1997–12.2002	Finland, Helsinki	Retrospective cohort study	EMS cardiac arrest registry.	Non-specified age; witnessed arrest; cardiac etiology; ventricular fibrillation.	From 2000 compression only. Standard BLS if suspected hypoxia.	9 (Selection 4; Comparability 2; Outcome 3)
Lewis et al., 2013 [42]	01.2011–12.2011	USA, Washington, King County	Retrospective cohort study	King County EMS Department records.	Adults; cardiac arrest recognised by dispatcher. Excluded: If traumatic etiology; if arrest in a medical facility; if emergency call handled by a nonparticipating dispatch center.	Compression only.	8 (Selection 4; Comparability 1; Outcome 3)
Oman et al., 2016 [43]	01.2011–12.2012	Ireland, regional centre	Retrospective cohort study	OHCAR (Out-of-Hospital Cardiac Arrest Registry).	Adult and paediatric; bystander next to the patient.	Not specified.	7 (Selection 4; Comparability 0; Outcome 3)
Park et al., 2018 [44]	01.2012–12.2015	Korea, nationwide	Retrospective cohort study	EMS cardiac arrest & dispatcher CPR registry.	Adult; cardiac etiology. Excluded: If no EMS resuscitation effort; if missing information on neurological outcome at discharge.	Compression only.	8 (Selection 4; Comparability 1; Outcome 3)
Rea et al., 2001 [25]	1983–2000	USA, Washington, King County	Retrospective cohort study	Database of King County Emergency Services Division & patient records.	Adult; cardiac etiology.	Standard BLS starting with ventilations.	9 (Selection 4; Comparability 1; Outcome 3)
Shibahashi et al. 2019 [45]	01.2010–12.2016	Japan, nationwide	Retrospective cohort study	Fire and Disaster Management Agency’s (FDMA) nationwide registry.	Adult. Excluded: if transported by a physician-manned ambulance; if resuscitation was not attempted; if missing outcomes.	Not specified.	6 (Selection 4; Comparability 0; Outcome 2)
Viereck et al., 2017 [46]	01.2013–12.2013	Denmark, capital region	Retrospective cohort study	Danish Cardiac Arrest Registry, the Mobile Critical Care Unit database & Danish Civil Registration System.	Non-specified age; bystander CPR provided; EMS treated. Excluded: If missing information on or time for initiation of bystander CPR; if missing emergency call record.	Compression only. Standard BLS if trained bystander.	7 (Selection 4; Comparability 0; Outcome 3)
Wu et al., 2018 [47]	01.2011–12.2014	USA, Arizona	Retrospective cohort study	Save Hearts in Arizona Registry and Education (SHARE) Program.	Adult; cardiac etiology. Excluded: if arrest in medical facility; if a DNR order existed; if EMS witnessed, if missing emergency call record; if call was transferred from another agency.	Compression only.	8 (Selection 4; Comparability 1; Outcome 3)

Standard BLS includes all protocols including compressions and ventilations incl. protocols starting with ventilations and pediatric BLS

*BLS* basic life support, *CPR* cardiopulmonary resuscitation, *DNR* do not resuscitate, *EMS* emergency medical services, *OHCA* out-of-hospital cardiac arrest, *NOS* Newcastle-Ottawa Scale, *yo* years old

A total of 661,059 OHCA were included of which 114,284 (17%) received DA-CPR; Shibahashi et al. [45] accounted for 88% of all OHCA in the review (582,483) and 76% of arrests receiving DA-CPR (86,913) (Tables 3, 4 and 5). The percentage of OHCAs receiving DA-CPR varied from 2 to 65% between studies with a median of 38% and IQR of 25 to 43%. Dispatcher instructions resulted in bystander CPR in 59–100% of the dispatchers attempts, median 70% (five studies) [17, 38, 40, 41, 43].

**Table 3:** Neurologically intact survival, Cerebral Performance Category 1–2, according to bystander CPR group

Author, year of publication	CPC 1 or 2 at discharge			CPC 1 or 2 at one-month			CPC 1 or 2 at six-month			Adjusted OR (CI) for CPC at …	Proportion of OHCA calls where DA-CPR was provided % (m/M)
	DA-CPR provided % (n/N)	Bystander CPR provided without DA % (n/N)	No bystander CPR (n/N)	DA-CPR provided % (n/N)	Bystander CPR provided without DA % (n/N)	No bystander CPR % (n/N)	DA-CPR provided % (n/N)	Bystander CPR provided without DA % (n/N)	No bystander CPR % (n/N)		
Chang et al., 2018 [37]	5.1% (38/747)	7.5% (22/293)	1.5% (15/980)							…discharge:^e^ No BCPR: reference BCPR: 3.32 (1.38–7.97) DA-CPR: 2.82 (1.25–6.38)	37.0% (747/2020)
Goto et al., 2014 [38]				3.7% (74/2019)^a^	6.3% (44/703)	2.5% (57/2287)				…one-month:^f^ No BCPR: reference BCPR: 1.68 (1.07–2.62) DA-CPR: 1.81 (1.24 to 2.67)	40.3% (2019/5009)
Harjanto et al., 2016^b^ [39]				0% (0/52)	4.2% (32/769)	1.6% (34/2147)					1.8% (52/2968)
Hiltunen et al., 2015 [40]							26.4% (14/53)	22.9% (11/49)	21.2% (7/33)		39.3% (53/135)
Lewis et al., 2013 [42]	16.7% (35/210)		16.0% (15/94)								44.1% (210/476)
Park et al., 2018 [44]	5.0% (961/19,343)	5.7% (384/6753)	2.2% (605/27,144)							…discharge: ^g^ Rural areas: No BCPR: reference BCPR: 2.56 (1.23–5.32) DA-CPR: 3.53 (1.84–6.77) Urban areas: No BCPR: reference BCPR: 1.37 (1.18–1.60) DA-CPR: 1.59 (1.41–1.79)	36.3% (19,343/53,240)
Shibahashi et al., 2019 [45]				3.1% (2687 /86,913)^c^	5.7% (2682 /46,964)^c^	2.6% (11,660 /448,606)^c^				…one-month:^h^ No BCPR^c^: reference BCPR^c^: 2.25 (2.15–2.36) DA-CPR^c^: 1.30 (1.24–1.36)	14.9% (86,913/582,483)^c^
Wu et al., 2018 [47]	8.8% (85/963)	12.9% (68/527)	6.6% (48/731)							…discharge: ^i^ No BCPR: reference BCPR: 1.58 (1.05–2.39) DA-CPR: 1.56 (1.06–2.31)	43.4% (1002/2310)^d^

*BCPR* bystander CPR, *CI* 95% confidence interval, *CPC* cerebral performance category, *CPR* cardiopulmonary resuscitation, *DA* dispatcher assistance, *DA-CPR* dispatcher assisted CPR, *EMS* emergency medical services, *m* number of OHCAs who received DA-CPR, *M* total number of OHCAs, *n* number with CPC 1 or 2, *N* total number of OHCAs, *OHCA* out-of-hospital cardiac arrest, *OR* odds ratio

^a^CPC 1–2 at one-month when DA was offered but bystander didn’t provide CPR: 3.1% (83/2698). CPC 1–2 at one-month in cases where DA was not offered: 3.0% (48/1608)

^b^Neurologically intact survival at discharge or at day 30 after arrest

^c^“DA-CPR provided” was defined as CPR initiated by DA. The “bystander CPR provided without DA”-group included 39.1% calls where DA was provided but CPR started prior to DA. The “no bystander CPR”-group included 32.0% calls where DA was given but no bystander CPR was provided

^d^Missing data on survival, why the numbers in the three survival data columns do not add up to 1002 and 2310 respectively

^e^Adjusted for: age, sex, etiology of arrest, witness status, shockable rhythm at scene, location of arrest, response time interval, and level of property value of a community

^f^Adjusted for: age, sex, presumed cardiac etiology, witnessed by a family member, shockable initial rhythm, and call-to-response time

^g^Adjusted for: age, sex, witnessed status, initial rhythm at scene, location of arrest, EMS response time, and interaction term: bystander CPR x rural/urban location

^h^Adjusted for: age, sex, presumed cardiac etiology, witnessed status, initial shockable rhythm, call-to-response time, call-to-hospital time, and period of hospital admittance

^i^Adjusted for: age, sex, witnessed status, location of arrest, EMS arrival time

**Table 4:** ROSC according to bystander CPR group

Author, year of publication	ROSC			Adjusted OR (CI) for ROSC at …	Proportion of OHCA calls where DA-CPR was provided% (m/M)
	DA-CPR provided % (n/N)	Bystander CPR provided without DA% (n/N)	No bystander CPR% (n/N)		
Chang et al., 2018^a^ [37]	6.6% (49/747)	9.9% (29/293)	3.3% (32/980)		37.0% (747/2020)
Harjanto et al., 2016^c^ [39]	26.9% (14/52)	30.3% (233/769)	27.2% (583/2147)		1.8% (52/2968)
Hiltunen et al., 2015^d^ [40]	66.0% (35/53)	61.2% (30/49)	54.5% (18/33)		39.3% (53/135)
Park et al., 2018^a^ [44]	7.4% (1433/19,343)	8.9% (599/6753)	3.8% (1030/27,144)		36.3% (19,343/53,240)
Shibahashi et al., 2019^b^ [45]	9.2% (8017/86,913)	13.6% (6392/46,964)	8.1% (36,423/448,606)	No BCPR: reference^f^ BCPR: 1.76 (1.71–1.81) DA-CPR: 1.34 (1.31–1.38)	14.9% (86,913/582,483)
Viereck et al., 2017^b^ [46]	33.8% (118/357)	41.2% (77/191)		DA-CPR: reference^g^ BCPR: 0.88 (0.56–1.38)	65.1% (357/548)
Wu et al., 2018^d^ [47]	16.0% (154/964)	20.9% (112/535)	15.1% (111/737)		43.4% (1002/2310)^e^

*CPR* cardiopulmonary resuscitation, *DA* dispatcher assistance, *DA-CPR* dispatcher assisted CPR, *n* number with ROSC, *N* total number of OHCAs, *m* number of OHCAs who received DA-CPR, *M* total number of OHCA in the study population, *OHCA* out-of-hospital cardiac arrest, *ROSC* return of spontaneous circulation

^a^ROSC at hospital arrival

^b^ROSC before hospital arrival

^c^ROSC prehospitally or in the emergency department

^d^Sustained ROSC

^e^Missing data on survival, why the numbers in the three survival data columns do not add up to 1002 and 2310 respectively

^f^Adjusted for: age, sex, presumed cardiac etiology, witnessed status, initial shockable rhythm, call-to-response time, call-to-hospital time, and period of hospital admittance

^g^Adjusted for: age, sex, witnessed status, and number of bystanders

**Table 5:** Survival according to bystander CPR group

Author, year of publication	Survival at discharge			Survival at one-month			Survival at one-year			Adjusted OR (CI) for survival at …	Proportion of OHCA calls where DA-CPR was provided % (m/M)
	DA-CPR provided % (n/N)	Bystander CPR provided without DA % (n/N)	No bystander CPR % (n/N)	DA-CPR provided % (n/N)	Bystander CPR provided without DA % (n/N)	No bystander CPR % (n/N)	DA-CPR provided % (n/N)	Bystander CPR provided without DA % (n/N)	No bystander CPR % (n/N)		
Chang et al., 2018 [37]	8.6% (64/747)	13.0% (38/293)	3.5% (34/980)							…discharge:^f^ No BCPR: reference BCPR: 2.87 (1.57–5.25) DA-CPR: 2.23 (1.33–3.74)	37.0% (747/2020)
Eisenberg et al., 1985 [17]	20.7% (12/58)	24.7% (21/85)	13.4% (15/112)								22.7% (58/255)
Goto et al., 2014 [38]				11.5% (232/2019)^a^	14.9% (105/703)	8.4% (191/2287)				…one-month:^g^ No BCPR: reference BCPR: 1.62 (1.23–2.11) DA-CPR: 1.63 (1.32–2.02)	40.3% (2019/5009)
Harjanto et al., 2016 [39]				1.9% (1/52)^b^	6.2% (48/769)	3.1% (67/2147)					1.8% (52/2668)
Hasselqvist et al., 2015 [7]				10.9% (167/1530)	15.4% (374/2427)						38.7% (1530/3957)
Hiltunen et al., 2015 [40]	45.3% (24/53)	46.9% (23/49)	39.4% (13/33)				32.1% (17/53)	40.8% (20/49)	21.2% (7/33)		39.3% (53/135)
Kuisma et al., 2005 [41]	61.6% (53/86)^c^										29.4% (86/346)
Lewis et al., 2013 [42]	18.1% (38/210)		17.0% (16/94)								44.1% (210/476)^d^
Oman et al. 2016 [43]	4.3% (2/47)	15.4% (2/13)	4.5% (1/22)								57.3% (47/82)
Park et al., 2018 [44]	7.1% (1381/19,343)	9.0% (608/6753)	4.7% (1285/27,144)							…discharge: ^h^ No BCPR: reference **Rural areas:** BCPR: 1.75 (1.01–3.04) DA-CPR: 2.15 (1.35–3.43) **Urban areas:** BCPR: 1.15 (1.02–1.30) DA-CPR: 1.10 (1.01–1.20)	36.3% (19,343/53,240)
Rea et al., 2001 [25]	15.2% (283/1867)	21.4% (470/2193)	11.3% (361/3205)							…discharge: ^i^ No BCPR: reference BCPR: 1.69 (1.42–2.01) DA-CPR: 1.45 (1.21–1.73)	25.7% (1867/7265)
Shibahashi et al., 2019 [45]				5.6% (4834/86,913)	8.7% (4102/46,964)	5.0% (22,450/448,606)				…one-month:^j^ No BCPR: reference BCPR: 1.76 (1.69–1.82) DA-CPR: 1.24 (1.19–1.28)	14.9% (86,913/582,483)
Viereck et al., 2017 [46]				16.7% (56/357)	27.2% (49/191)					…one-month:^k^ DA-CPR: reference BCPR: 1.14 (0.68–1.92)	65.1% (357/548)
Wu et al., 2018 [47]	12.3% (120/973)	15.5% (83/534)	8.6% (63/735)							…discharge: ^l^ No BCPR: reference BCPR: 1.51 (1.04–2.18) DA-CPR: 1.64 (1.16–2.30)	43.4% (1002/2310)^e^

*BCPR* bystander CPR, *CI* 95% confidence interval, *CPC* cerebral performance category, *CPR* cardiopulmonary resuscitation, *DA* dispatcher assistance, *DA-CPR* dispatcher assisted CPR, *EMS* emergency medical services, *m* number of OHCAs who received DA-CPR, *M* total number of OHCAs, *n* number with CPC 1 or 2, *N*, total number of OHCAs, *OHCA* out-of-hospital cardiac arrest, *OR* odds ratio

^a^Survival at one-month when DA was offered but bystander didn’t provide CPR: 10.3% (279/2698). Survival at one-month in cases where DA was not offered: 9.0% (144/1608)

^b^Survival to discharge or at day 30 after arrest

^c^Survival when DA was not offered: 32.3% (72/223), includes cases where bystander provided CPR without DA. Survival when DA was offered: 43.1% (53/123), includes both cases with DA-CPR provided and no bystander CPR

^d^Includes calls with bystander CPR without DA and calls where OHCA was not recognized, why the numbers in the three survival columns don’t add up to 476

^e^Missing data why the numbers in the three survival columns does not add up to 1002 and 2310

^f^Adjusted for: age, sex, etiology of arrest, witness status, shockable rhythm at scene, location of arrest, response time interval, level of property value of community

^g^Adjusted for: age, sex, presumed cardiac etiology, witnessed by family member, shockable initial rhythm, call-to-response time

^h^Adjusted for: age, sex, witnessed status, initial rhythm at scene, location of arrest, EMS response time, interaction term: bystander CPR x rural/urban location

^i^Adjusted for: age, sex, witnessed status, location of arrest, EMS response time

^j^Adjusted for: age, sex, presumed cardiac etiology, witnessed status, initial shockable rhythm, call-to-response time, call-to-hospital time, period of hospital admittance

^k^Adjusted for: age, sex, witnessed status, number of bystanders

^l^Adjusted for: age, sex, witnessed status, location of arrest, EMS arrival time

Two studies reported data on a paediatric population only [37, 38], five studies reported data on an adult population only [25, 42, 44, 45, 47], and seven studies reported on a mixed population [7, 17, 39–41, 43, 46].

Concerning predictors of survival, six studies included only arrests of cardiac etiology [17, 25, 39, 41, 44, 47], three studies included only witnessed arrests [7, 40, 41], two included only arrests with initial shockable rhythm [40, 41],

Of studies including both witnessed and unwitnessed arrest, witnessed arrest was more common in the bystander-initiated CPR group than in the DA-CPR group (seven studies) [25, 37, 38, 44–47]. A higher rate of initial shockable rhythm was also reported for bystander–initiated CPR than for DA-CPR (six studies) [37, 38, 44, 45, 47]. Finally, the bystander-initiated group had more arrests that occurred in a public location, whereas a residential location was more common in the DA-CPR group (five studies) [25, 37, 44, 46, 47]. The no bystander CPR group had the lowest number of arrests that were witnessed and with initial shockable rhythm (eight studies) [25, 37, 38, 42, 44–47]. Some studies reported that this group had a similar rate of arrests in a public location as in the bystander-initiated group, while others reported a slightly lower rate (six studies) [25, 37, 42, 44, 46, 47].

A standardized protocol for DA-CPR instructions existed in all studies (Table 2). The protocol demanded compression-only BLS in seven studies [39–42, 44, 46, 47]. Common exceptions for compression-only included OHCA in children, suspected hypoxia, or when bystander was trained.

### Risk of bias within studies

The included studies were awarded from 6 to 9 stars on the Newcastle-Ottawa Scale (Table 2). The most common reason for increased risk of bias according to the Newcastle-Ottawa Scale (lower score) was that the study did not control for confounding factors.

### Results of individual studies

**Fig. 2:**
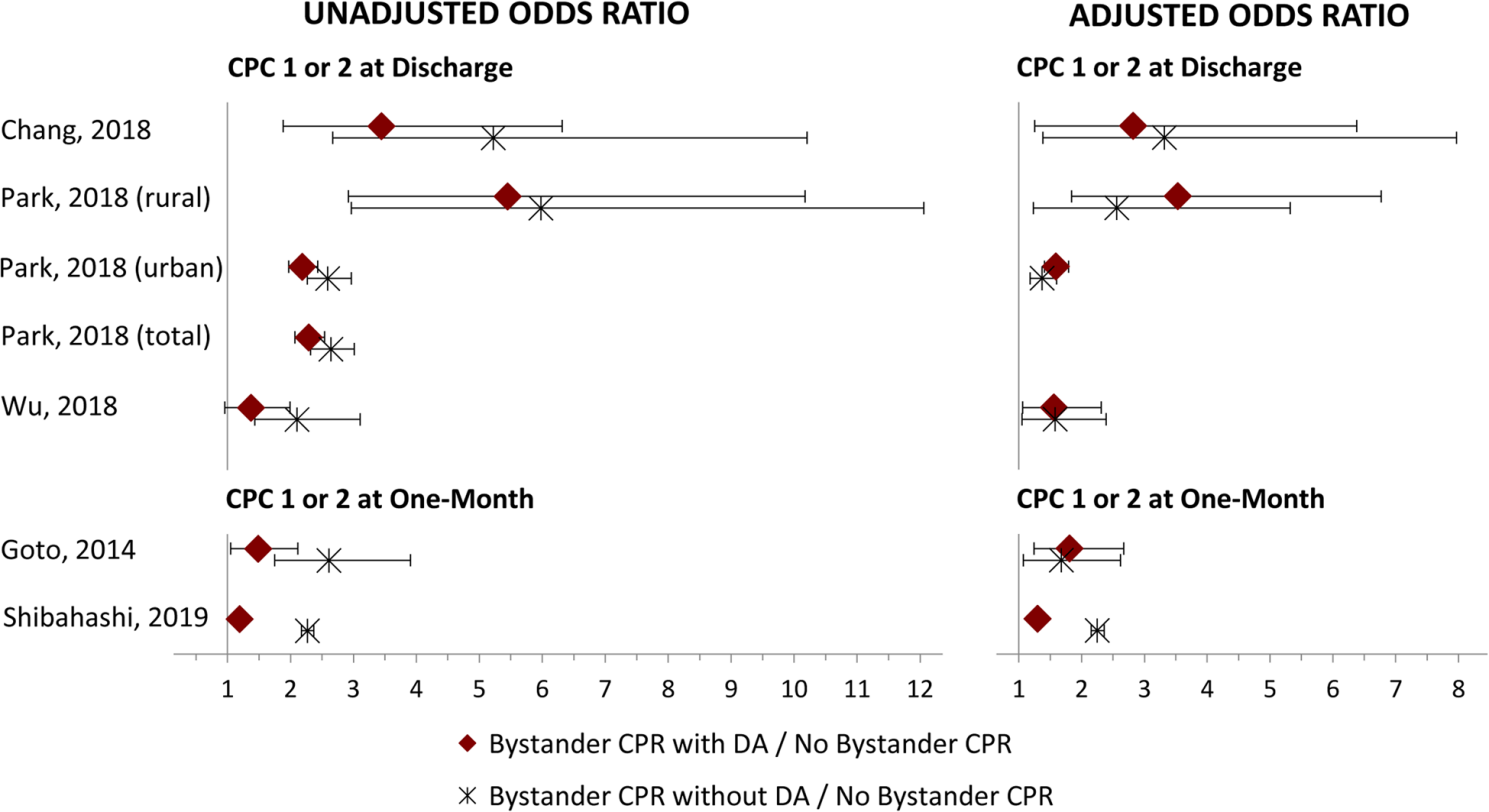
Forest plot of the unadjusted and adjusted neurologically intact survival outcomes at discharge and one-month. Red diamond represents the unadjusted/adjusted odds ratio of bystander CPR with DA with no bystander CPR as reference. Black star represents unadjusted/adjusted odds ratio between bystander CPR without DA and no bystander CPR. Black lines are confidence intervals. NB. The individual studies have adjusted for different factors. Details are provided in Table 3. CPC, Cerebral Performance Category; CPR, cardiopulmonary resuscitation; DA, dispatcher assistance

Results of individual studies are presented in Tables 3, 4, and 5 and in Fig. 2, which show that generally, the higher odds for neurologically intact survival with bystander-initiated CPR without dispatcher assistance compared with DA-CPR were reduced when adjusting for confounding factors.

### Summary measures

**Fig. 3:**
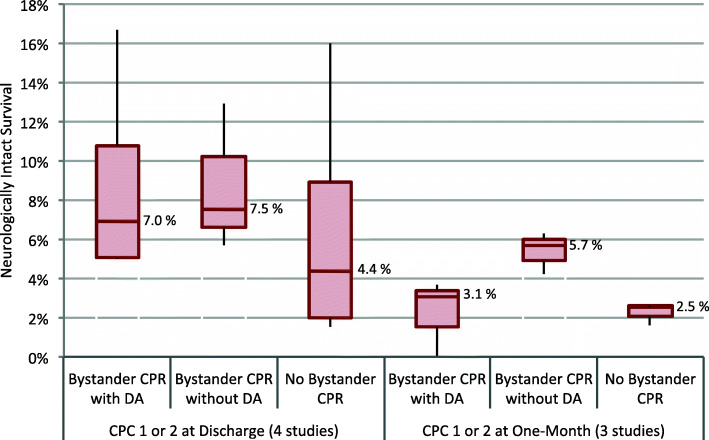
Median neurologically intact survival defined as Cerebral Performance Category (CPC) 1 or 2 at hospital discharge or one-month with interquartile range (IQR) and minimum/maximum range according to bystander CPR status. Horizontal line within boxes represents median, upper and lower border of boxes reflect IQR, and the black lines show the range of the observations. Details are reported in Table 3. DA, dispatcher assistance; CPC, Cerebral Performance Category; CPR, cardiopulmonary resuscitation

Median neurologically intact survival at discharge with DA-CPR was 7.0% (IQR: 5.1–10.8%), with bystander-initiated CPR without dispatcher assistance 7.5% (IQR: 6.6–10.2%), and with no provision of bystander CPR before EMS arrival 4.4% (IQR: 2.0–9.0%) (four studies, Fig. 3, Table 3) [37, 42, 44, 47]. At one-month, median neurologically intact survival was 3.1% (IQR: 1.6–3.4%) with DA-CPR, 5.7% (IQR: 5.0–6.0%) with bystander-initated CPR, and 2.5% (IQR: 2.1–2.6%) with no bystander CPR (three studies, Fig. 3, Table 3) [38, 39, 45]. Only one study reported neurologically intact survival at longer follow-up (six-months) [40].

When excluding studies with only pediatric patients (2 studies), median neurologically intact survival at discharge with DA-CPR was 8.8% (IQR: 6.9–12.8%), with bystander-initiated CPR without dispatcher assistance 9.3% (IQR: 7.5–11.1%), and with no bystander CPR 6.6% (IQR: 4.4–11.3%) [42, 44, 47]. At one-month, the numbers were 1.6% (IQR: 0.8–2.3%) for DA-CPR, 5.0% (IQR: 4.6–5.3%) with bystander-initiated CPR, and 2.1% (IQR: 1.9–2.4%) with no bystander CPR [39, 45].

Eleven out of the 33 eligible studies met the additional criteria of *witnessed OHCA.* Of these 11 studies, three did not provide data on neurologically intact survival, and after removing studies with overlapping study populations four studies remained (Table 2 and Supplementary Material Table 1). Lee et al. [49] reported neurologically intact survival to discharge; DA-CPR: 8.8%; bystander-initiated CPR 10.1%; no bystander CPR 4.2%. Shimamoto et al. [50] and Takei et al. [26] reported neurologically intact survival at one-month; DA-CPR: 6.0% & 4.5%; bystander-initiated CPR: 8.3% & 6.0%; no bystander CPR: 3.4% & 3.1%. Hiltunen et al. [40] reported for a 6-months follow-up (Table 3).

For the secondary outcomes, median ROSC was 16.0% (IQR: 8.3–30.4%) in the DA-CPR group, 20.9% (IQR: 11.8–35.8%) in the bystander-initiated CPR group, and 11.6% (IQR: 4.9–24.2%) in the no bystander CPR group (seven studies, Table 4) [37, 39, 40, 44–47].

**Fig. 4:**
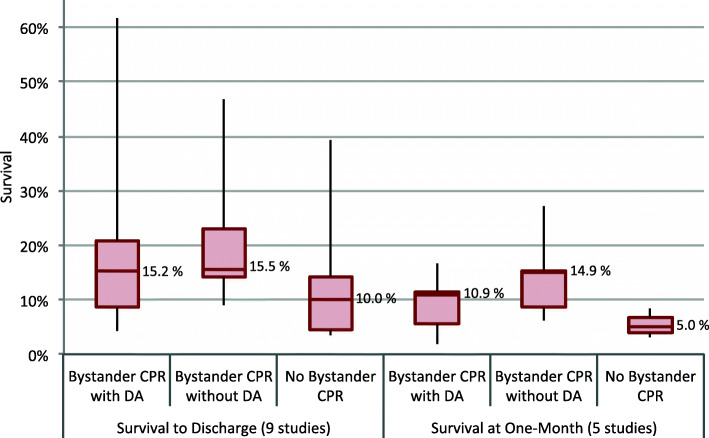
Median survival to hospital discharge or one-month survival with interquartile range (IQR) and minimum/maximum range according to bystander CPR status. Horizontal line within boxes represents median, upper and lower border of boxes reflect the IQR, and the black lines show the range of the observations. Details are provided in Table 5. DA, dispatcher assistance; CPR, cardiopulmonary resuscitation

Median survival to hospital discharge with DA-CPR was 15.2% (IQR: 8.6–20.7%), with bystander-initiated CPR survival was 15.5% (IQR: 14.2–23.1%), and with no bystander CPR 10.0% (IQR: 4.7–14.3%) (nine studies, Fig. 4, Table 5) [17, 25, 37, 40–44, 47]. At one-month median survival rate was 10.9% (IQR: 5.6–11.5%) with DA-CPR, 14.9% (IQR: 8.7–15.4%) with bystander-initated CPR, and 5.0% (IQR: 4.1–6.7%) with no bystander CPR (five studies, Fig. 4, Table 5) [7, 38, 39, 45, 46].

## Discussion

This systematic review generates a broad perspective of the effect of DA-CPR on neurologically intact survival in patients with OHCA by including 14 studies from a variety of different EMS systems. Both DA-CPR and bystander-initiated CPR without dispatcher assistance improve neurologically intact survival compared with no bystander CPR. However, DA-CPR shows inferior outcomes compared with bystander CPR without dispatcher assistance. This may be partly accounted for by an imbalanced distribution of confounders like witnessed arrest between groups, which is indicated by the reduced difference when adjusting for confounding factors. The same trends among the three groups are observed for the secondary outcomes: prehospital ROSC or ROSC at hospital arrival and survival at discharge or one-month and longer. That is, higher survival rate and rate of ROSC with DA-CPR and bystander-initiated CPR than with no bystander CPR.

### Dispatcher-assisted CPR versus no bystander CPR

This systematic review suggests that DA-CPR increases the rate of neurologically intact survival compared with no bystander CPR. Odds ratios adjusted for established confounders that compared DA-CPR with no bystander CPR are provided by five studies. They all report significantly higher odds of neurologically intact survival with DA-CPR [37, 38, 44, 45, 47].

Previous studies demonstrate the effect of immediate bystander CPR, which slows the inevitable decline in survival until defibrillation and advanced EMS treatment [5, 51]. The studies included in this review show an increase in ROSC with DA-CPR compared with no bystander CPR. Thus, the prehospital intervention is strengthened by the dispatchers and their role in motivating and assisting bystanders to provide early CPR. Also, survival rates are higher with DA-CPR than no bystander CPR for all follow-up periods reported.

### Bystander CPR with and without dispatcher assistance

DA-CPR appears inferior to bystander-initiated CPR without dispatcher assistance looking at the crude neurologically intact survival outcomes, as well as ROSC and survival. However, when adjusting for confounding factors the difference is reduced, and confounding factors account for some of the difference in the crude neurologically intact survival outcomes between the DA-CPR group and bystander-initiated CPR group. For example, witnessed arrest and initial-shockable rhythm, two established predictors of survival, are both seen more frequently in the bystander-initiated CPR group than in the DA-CPR group [36]. It is likely that time to first compression is shorter for bystander-initiated CPR, because a trained bystander can recognize and initiate CPR without the delay of the emergency call, during which the dispatcher first has to recognize cardiac arrest and secondly provide instructions on CPR [46]. A trained bystander initiating CPR reduces time to first compression and increases time with CPR until EMS arrival compared with an untrained bystander relying on dispatcher assistance; time factors that are associated with higher survival rates [7, 24–26].

Simulation studies have demonstrated comparable quality of DA-CPR and bystander CPR without dispatcher assistance [27, 28]. Whether the results are transferable to real-life OHCA resuscitation attempts has not yet been proven, and differences in CPR quality may account for differences in outcomes. Finally, most of the simulation studies have investigated the quality of CPR among young adults while the majority of cardiac arrests occur in men above 60 at home [13]. This leaves CPR to his elderly wife, and physical limitation is a frequent reason for bystanders to reject CPR [52].

### Definition of DA-CPR

Little consensus exists on how to define and report DA-CPR [33, 53]. This means that variability in extent and quality of dispatcher instructions, and the extent and quality of the bystanders’ CPR attempt will influence OHCA outcomes and may produce inter-study variability. For example, none of the included studies specified what motivation and instruction the dispatcher needed to provide in order to define the bystander attempt as dispatcher-assisted. Also, information defining the actions bystanders were to provide and how the provision of bystander CPR was determined varied and sometimes lacked. In some studies bystander CPR was determined from the emergency call, while in other studies from the report of on-site EMS personnel. Both methods have limitations, i.e. Linderoth et al. [54] found that the understanding of bystander interventions from emergency call records was not always accurate (assessed emergency call records versus closed-circuit televisions from public locations).

The outcomes in OHCAs with dispatcher assistance reported in this review reflect cases where the bystander initiated CPR following instructions. Not every attempt by the dispatcher results in bystander CPR, which is further discussed in Supplementary Material 2.

### Strengths, limitations, and risk of bias

This review focuses on neurologically intact survival, a marker for not only survival but survival with preserved neurologic function and quality of life. A systematic, recent and comprehensive literature search was performed using broad search criteria to report contemporary knowledge and minimize selection bias. Study characteristics and results of individual studies are presented along with any study-reported adjusted outcomes for transparency incl. transparency of the inter-study variability. Summary measures are provided to condense contemporary knowledge.

#### Limitations at study level

CPR status (DA-CPR, bystander-initiated CPR without assistance, no bystander CPR) was determined from emergency calls and from reports of the dispatchers and EMS personnel. These may not be accurate. Two studies determined bystander CPR status from either a follow-up telephone interview with the bystander or interviewing the bystanders before leaving the scene [17, 38], three determined it from the emergency call recording as well as observations by the EMS on scene [25, 39, 47], three from only EMS observations on scene [40, 44, 45], and one study only from the emergency call recording [42]. The remaining six studies did not specify.

All studies are observational with an inherent risk of selection bias. Some studies attempted to control predictors of survival by study design, in the inclusion criteria, and/or afterwards in data analyses with regression models but there was no uniform reporting.

It is generally accepted that publication bias exists and that studies with large effects have the highest chances of being published [55].

#### Limitations at review level

This review excluded 14 studies because of overlapping data. The criteria for inclusion in case of overlapping data were: 1) reporting primary outcomes, 2) reporting secondary outcomes, and 3) the largest population. These were chosen to ensure data on relevant outcomes and because a larger population limits the risk of publication bias, the influence of outliers, and the accumulation of OHCAs with either many or few risk factors in the different intervention groups by chance [55]. Other selection criteria could affect the results of the review.

This review defined PICOS to include all cardiac arrests. This was chosen to reduce selection bias because inclusion criteria of studies tend to differ between publications from different EMS systems. Of the 33 studies eligible for this review, all European-based studies (6 studies) would have been ineligible if the study population had been defined as *only adult OHCAs* because these studies did not apply an age criterium. The heterogeneous inclusion criteria of the included studies could reduce the precision of the results of the review. Neither did the review differentiate between dispatcher protocols, i.e. provision of compression-only versus standard BLS instructions nor between definitions of DA-CPR, which were often vague. Both factors introduce variability and heterogeneity in the DA-CPR group across the included studies, and higher survival rates with compression-only instructions have been shown [56–60].

The studies reported different rates of OHCAs receiving DA-CPR of the total OHCAs. This may not only reflect the success rate but also how often dispatchers attempt to instruct bystanders in CPR. If dispatcher instructions are attempted only in OHCAs with a high probability of survival, the survival rate and effect of DA-CPR appear higher. In the systematic review on OHCA incidences by Berdowski et al. 2010, Asia had the highest percentage of EMS treated OHCA while survival lacked behind [4]. One explanation may be a lower threshold for initiating CPR, also for patients who have been in cardiac arrest for a long time.

Finally, the study by Shibahashi et al. [45] accounted for 88% of all OHCA and 76% of arrests receiving DA-CPR included in the review. To mitigate dominance by one study and improve transparency, individual data were presented in tables and forest plots, and median and IQR were used as summary measures.

Contributions to literature are discussed in Supplementary Material 2.

### Perspective

In future studies, it may be relevant to assess the effect of DA-CPR in untrained vs. trained bystanders as training status may confound the results of the current publications. Also, the DA-CPR protocol should likely be adjusted to the training status of the bystanders and the setting and situation of the OHCA, why a “one-size-fits-all” protocol may not exist. New technological solutions such as live video streaming from the OHCA location transmitted via the bystander’s smartphone to the dispatcher may enhance communication and improve the quality of the DA-CPR.

## Conclusions

This systematic review shows that DA-CPR and bystander-initiated CPR without dispatcher assistance increased neurologically intact survival at discharge and one-month compared with no bystander CPR. Also, the survival rate at discharge and one-month as well as the rate of ROSC are higher with DA-CPR and bystander-initiated CPR compared with no bystander CPR. The crude outcome parameters indicate that bystander-initiated CPR without dispatcher assistance is superior to DA-CPR but this difference is greatly reduced when adjusting for confounding factors.

## Supplementary Information

**Additional file 1: Supplementary Material Table 1.** Study Characteristics of Studies Excluded due to Population Overlap.

**Additional file 2: Supplementary Material 2. **DA-CPR Attempts and Barriers of Dispatcher Assistance.

**Additional file 3:** PRISMA Checklist.

## Data Availability

Data derived from published peer-reviewed articles.

## Abbreviations

BLS: Basic life support
CPC: Cerebral Performance Category
CPR: Cardiopulmonary resuscitation
DA-CPR: Dispatcher-assisted cardiopulmonary resuscitation
EMS: Emergency medical services
FF: Fredrik Folke (co-author)
FL: Freddy Lippert (co-author)
GL: Gitte Linderoth (reviewer 2)
IQR: Interquartile range
KEE: Kristine Elisabeth Eberhard (reviewer 1)
MTG: Mads Christian Tofte Gregers (reviewer 2)
OHCA: Out-of-hospital cardiac arrest
PICOS: Population, intervention, comparison, outcomes and study
PRISMA: Preferred Reporting Items for Systematic Reviews and Meta-Analyses
ROSC: Return of spontaneous circulation
