# Supplementary material for: Impact of dispatcher-assisted cardiopulmonary resuscitation on neurologically intact survival in out-of-hospital cardiac arrest: a systematic review
Source: Scand J Trauma Resusc Emerg Med. 2021 May 24;29:70. doi: 10.1186/s13049-021-00875-5 (PMC8147398; doi:10.1186/s13049-021-00875-5)
Supplement: Supplementary file 1 — Additional file 1: Supplementary Material Table 1. Study Characteristics of Studies Excluded due to Population Overlap. [file 13049_2021_875_MOESM1_ESM.docx]

**SUPPLEMENTARY MATERIAL TABLE 1: Study Characteristics of Studies Excluded due to Population Overlap**

| **Author, year of publication** | **Study period** | **Location** | **Study design** | **Data source**  **/Name of registry** | **Population / Inclusion criteria** | **Proportion of OHCA calls where DA-CPR was provided % (m/M)** | **Overlap with xx**  **included study  / Reason for exclusion** |
| --- | --- | --- | --- | --- | --- | --- | --- |
| Akahane et al., 2012^1^ | 01.2005 – 12.2008 | Japan,  nationwide | Retrospective  cohort study | Fire and Disaster Management Agency's (FDMA) nationwide registry | Paediatric (<20 yo); witnessed arrests; EMS arrival ≤60 min. Excluded: If unknown etiology; if bystander shocked with AED. | 505*/1780  *DA offered | Goto et al., 2014 /Smaller population |
| Bång et al., 1999^2^ | 01.1994 – 03.1996 | Sweden, Gothenburg | Prospective cohort study | Prospective  collection | Non-specified age; cardiac arrest  on arrival of ambulance. | 142/427 | Hasselqvist et al., 2015  /Not reporting primary outcome |
| Chang et al., 2018^3^ | 01.2012 – 12.2016 | Korea,  nationwide | Retrospective  cohort study | Korean OHCA registry | Paediatric (≤18yo).  Excluded: If <1yo; if EMS arrival ≥ 30 min. | 678/1953 | Chang et al., 2018  /Smaller population |
| Culley et al., 1991^4^ | 1988 | USA,  Washington, King County | Retrospective  cohort study | OHCA surveillance database & audio recordings | Adult; witnessed arrest;  shockable rhythm.  Excluded: If traumatic etiology or if arrest at medical facility. | 267/7227 | Rea et al., 2001  /Not reporting primary outcome |
| Fujie et al., 2014^5^ | 01.2004 – 12.2009 | Japan,  Ibaraki region | Retrospective cohort study | Fire and Disaster Management Agency's (FDMA) nationwide registry | Adult and paediatric;  non-traumatic etiology. | 298*/559  *DA offered | Shibahashi et al., 2019 & Goto et al., 2014  /Not reporting secondary outcome measures |
| Hagihara et al., 2018^6^ | 01.2005 – 12.2014 | Japan,  nationwide | Retrospective  cohort study | Fire and Disaster Management  Agency's (FDMA) nationwide registry | ﻿ Adult; witnessed arrest; cardiac etiology; bystander provided chest compressions; EMS arrival ≤ 60 min; hospital arrival ≤120 min. Excluded: If treated with AED by a bystander; if ALS was provided by a  medical doctor. | 55,978/87,400 | Shibahashi et al., 2019 /Smaller population |
| Hatakeyama et al., 2020^7^ | 01.2009 – 12.2015 | Japan,  Osaka | Retrospective cohort study | Osaka Municipal Fire Department registry | Adult; medical etiology. Excluded: If no EMS resuscitation attemps. | 3103/10,925 | Shibahashi et al., 2019  /Smaller population |
| Japanese Circulation Society Resuscitation Science Study Group, 2013^8^ | 01.2006 – 12.2012 | Japan,  nationwide | Retrospective cohort study | Fire and Disaster Management Agency's (FDMA) nationwide registry | Adult; witnessed arrest; subgroup with cardiac etiology. Excluded: If ROSC after bystander AED shock; if missing information on bystander CPR technique or arrest rhythm on EMS arrival. | 47,694/78,150 | Shibahashi et al., 2019 /Smaller population |
| Kaneko et al., 2019^9^ | 2014-2016 | Japan,  Kumamoto | Retrospective cohort study | Prehospital records | All OHCA patients | 1132/1607 | Shibahashi et al., 2019 /Smaller population |
| Lee et al., 2019^10^ | 01.2012 – 12.2013 | Korea,  nationwide | Retrospective  cohort study | EMS cardiac arrest, dispatcher CPR & national OHCA registry | Paediatric (≤18yo).  Excluded if missing neurological outcome at discharge. | 235/1013 | Chang et al., 2018  /Smaller population |
| Lee et al., 2019^11^ | 01.2013-12.2016 | Korea, nationwide | Retrospective cohort study | Korean National OHCA registry, EMS run sheets, EMS cardiac arrest and dispatcher CPR registries | Adult (≥18yo), EMS treated, witnessed, cardiac etiology. Excluded: If no EMS resuscitation attempt; if EMS witnessed; if arrest in a primary care clinic; if defibrillation by layperson; if layperson unwitnessed OHCA, if missing information on time of ROSC and defibrillation. | 12,154/25,450 | Park et al., 2018  /Smaller population |
| Moriwaki et al., 2016^12^ | 09.2007 – 02.2010 | Japan,  Yokohama | Retrospective cohort study | Interviews with bystanders and EMS crew & hospital records | Non-specified age. | 373/803 | Shibahashi et al., 2019 & Goto et al., 2014 /Not reporting primary outcome measures |
| Park et al., 2018^13^ | 01.2013 – 12.2016 | Korea,  Seoul | Before–after study | Korean OHCA  registry | Adult (>15yo); cardiac etiology.  Excluded: If arrest at medical facility; if missing data on neurological outcome. | 5223/12,670 | Park et al., 2018 /Smaller population |
| Ro et al., 2017^14^ | 01.2012 – 12.2014 | Korea, nationwide | Retrospective  cohort study | EMS cardiac arrest, dispatcher CPR registry & national OHCA registry | Adult; cardiac etiology. Excluded: If arrest in medical facility; if missing information on location or neurological outcome at discharge. | 11,791/37,924 | Park et al., 2018  /Smaller population |
| Ro et al., 2016^15^ | 01.2012 – 12.2014 | Korea,  nationwide | Retrospective  cohort study | EMS cardiac arrest, dispatcher CPR  & national OHCA registry | Paediatric (≤18yo).  Excluded if missing information on neurological outcome at discharge. | 705/1529 | Chang et al., 2018 /Smaller population |
| Shah et al., 2017^16^ | 01.2014 –12.2015 | USA, 20 state-based registries & 60 additional communities | Retrospective  cohort study | CARES (Cardiac Arrest Registry to Enhance Survival) registry | Adult. | 372/3335 | Wu et al., 2018  /Not reporting  primary outcome measures |
| Shimamoto et al., 2020^17^ | 01.2013 – 12.2015 | Japan, nationwide | Retrospective cohort study | Fire and Disaster Management Agency's (FDMA) nationwide registry | Adult; medical origin, witnessed arrest. Excluded: If no resuscitation attempt; if arrest in a medical facility; if missing outcome or first documented heart rhythm. | 40,087/104,621 | Shibahashi et al. 2019,  /Smaller population |
| Takahashi et al., 2018^18^ | 01.2008 – 12.2012 | Japan,  nationwide | Retrospective cohort study | Fire and Disaster Management Agency's (FDMA) nationwide registry | Adult; witnessed arrest; cardiac etiology. Excluded: If bystander provided shock with AED; if missing data on arrest rhythm; if time to EMS arrival ≥30 min or hospital arrival ≥97 min. | 15,082/37,899 | Shibahashi et al., 2019  /Smaller population |
| Takei et al., 2016^19^ | 2007 – 2012 | Japan,  nationwide | Retrospective cohort study | Fire and Disaster Management Agency's (FDMA) nationwide registry | Adult and paediatric; witnessed  arrest. Excluded: If ALS was provided by a medical doctor or if unknown. | 58,326/90,068 | Shibahashi et al., 2019 & Goto et al., 2014  /Smaller population |

Studies excluded because of overlapping data. AED, automated external defibrillator; ALS, advanced life support; CPR, cardiopulmonary resuscitation; DA-CPR, dispatcher assisted CPR; EMS, emergency medical services; m, number of OHCAs who received DA-CPR; M, total number of OHCA in the study population; OHCA, out-of-hospital cardiac arrest; yo, years old.

1. Akahane M, Ogawa T, Tanabe S, Koike S, Horiguchi H, Yasunaga Y, et al. Impact of telephone dispatcher assistance on the outcomes of pediatric out-of-hospital cardiac arrest. Crit Care Med. 2012;40:1410–6.

2. Bång A. Evaluation of dispatcher-assisted cardiopulmonary resuscitation. Eur J Emerg Med. 1999;6:175–83.

3. Chang I, Lee SC, Shin S Do, Song KJ, Ro YS, Park JH, et al. Effects of dispatcher-assisted bystander cardiopulmonary resuscitation on neurological recovery in paediatric patients with out-of-hospital cardiac arrest based on the pre-hospital emergency medical service response time interval. Resuscitation. 2018;130:49–56.

4. Culley LL, Clark JJ, Eisenberg MS, Larsen MP. Dispatcher-assisted telephone CPR: Common delays and time standards for delivery. Ann Emerg Med. 1991;20:362–6.

5. Fujie K, Nakata Y, Yasuda S, Mizutani T, Hashimoto K. Do dispatcher instructions facilitate bystander-initiated cardiopulmonary resuscitation and improve outcomes in patients with out-of-hospital cardiac arrest? A comparison of family and non-family bystanders. Resuscitation. 2014;85:315–9.

6. Hagihara A, Onozuka D, Shibuta H, Hasegawa M, Nagata T. Dispatcher-assisted bystander cardiopulmonary resuscitation and survival in out-of-hospital cardiac arrest. Int J Cardiol. 2018;265:240–5.

7. Hatakeyama T, Kiguchi T, Kobayashi D, Nakamura N, Nishiyama C, Hayashida S, et al. Effectiveness of dispatcher instructions-dependent or independent bystander cardiopulmonary resuscitation on neurological survival among patients with out-of-hospital cardiac arrest. J Cardiol. 2020;75:315–22.

8. Japanese-Circulation-Society-Resuscitation-Science Study-Group. Chest-compression-only bystander cardiopulmonary resuscitation in the 30:2 compression-to-ventilation ratio era. Nationwide observational study. Circ J. 2013;77:2742–50.

9. Kaneko T, Tanaka H, Uezono K, Karashima R, Iwashita S, Irie H, et al. Dispatcher-assisted cardiopulmonary resuscitation improves the neurological outcomes of out-of-hospital cardiac arrest victims: A retrospective analysis of prehospitalisation records in Kumamoto city. Crit Care Shock. 2019;22:9–15.

10. Lee YJ, Song KJ, Shin S Do, Lee SC, Lee EJ, Ro YS, et al. Dispatcher-Assisted Cardiopulmonary Resuscitation Program and Outcomes After Pediatric Out-of-Hospital Cardiac Arrest. Pediatr Emerg Care. 2019 Aug;35:561–7.

11. Lee SY, Hong KJ, Shin S Do, Ro YS, Song KJ, Park JH, et al. The effect of dispatcher-assisted cardiopulmonary resuscitation on early defibrillation and return of spontaneous circulation with survival. Resuscitation. 2019;135:21–9.

12. Moriwaki Y, Tahara Y, Kosuge T, Suzuki N. The effect of telephone advice on cardiopulmonary resuscitation (CPR) on the rate of bystander CPR in out-of-hospital cardiopulmonary arrest in a typical urban area. Hong Kong J Emerg Med. 2016;23:220–6.

13. Park JH, Shin S Do, Ro YS, Song KJ, Hong KJ, Kim TH, et al. Implementation of a Bundle of Utstein Cardiopulmonary Resuscitation Programs to Improve Survival Outcomes after Out-of-Hospital Cardiac Arrest in a Metropolis: A Before and After Study. Resuscitation. 2018;130:124–32.

14. Ro Y, Shin S, Lee Y, Lee S, Song K, Ryoo H, et al. Effect of Dispatcher-Assisted Cardiopulmonary Resuscitation Program and Location of Out-of-Hospital Cardiac Arrest on Survival and Neurologic Outcome. Ann Emerg Med. 2017;69:52–61.

15. Ro Y, Shin S, Song K, Hong K, Ahn K, Kim D, et al. Effects of Dispatcher-assisted Cardiopulmonary Resuscitation on Survival Outcomes in Infants, Children, and Adolescents with Out-of-hospital Cardiac Arrests. Resuscitation. 2016;108:20–6.

16. Shah M, Bartram C, Irwin K, Vellano K, McNally B, Gallagher T, et al. Evaluating Dispatch-Assisted CPR Using the CARES Registry. Prehospital Emerg Care. 2018;22:222–8.

17. Shimamoto T, Kiyohara K, Matsuyama T, Kitamura T, Kiguchi T, Nishiyama C, et al. Impact of Bystander Cardiopulmonary Resuscitation and Dispatcher Assistance on Survival After Out-of-Hospital Cardiac Arrest Among Adult Patients by Location of Arrest. Int Heart J. 2020;61:46–53.

18. Takahashi H, Sagisaka R, Natsume Y, Tanaka S, Takyu H, Tanaka H. Does dispatcher-assisted CPR generate the same outcomes as spontaneously delivered bystander CPR in Japan? Am J Emerg Med. 2018;36:384–91.

19. Takei Y, Kamikura T, Nishi T, Maeda T, Sakagami S, Kubo M, et al. Recruitments of trained citizen volunteering for conventional cardiopulmonary resuscitation are necessary to improve the outcome after out-of-hospital cardiac arrests in remote time-distance area: A nationwide population-based study. Resuscitation. 2016;105:100–8.
