# Supplementary material for: Impact of dispatcher-assisted cardiopulmonary resuscitation on neurologically intact survival in out-of-hospital cardiac arrest: a systematic review
Source: Scand J Trauma Resusc Emerg Med. 2021 May 24;29:70. doi: 10.1186/s13049-021-00875-5 (PMC8147398; doi:10.1186/s13049-021-00875-5)
Supplement: Supplementary file 2 — Additional file 2: Supplementary Material 2. DA-CPR Attempts and Barriers of Dispatcher Assistance. [file 13049_2021_875_MOESM2_ESM.docx]

**SUPPLEMENTARY MATERIAL 2**

**DA-CPR Attempts and Barriers of Dispatcher Assistance**

The outcomes in OHCAs with dispatcher assistance reported in this review reflect cases where the bystander initiated CPR following instructions. Not every attempt by the dispatcher results in bystander CPR. A median of 70% of cases where the dispatcher provided instructions resulted in bystander CPR with a range from 59-100%.^1–5^ Goto et al. 2014 reported a 3.1% neurologically intact survival rate at one-month for OHCAs where the dispatcher offered instructions versus 3.7% when bystander CPR was actually provided following the instructions. Kuisma et al. 2006 reported that 43% survived to discharge when the dispatcher offered instructions versus 62% when the bystander actually provided CPR following the instructions.

Several studies have reported barriers impeding DA-CPR: The most common barriers reported are physical limitations, emotional distress, or simply the rejection of instructions.^6,7^ Another major challenge, preceding dispatcher instructions, is recognition of cardiac arrest because failure of timely recognition delays interventions and reduces survival.^8,9^ Barriers may explain why only a few of the included studies reach more than 50% DA-CPR rates of the total OHCAs.

**Contributions to literature**

The findings of this review are in agreement with the previous systematic reviews by Bohm et al. 2011 and Nikolaou et al. 2019.^10,11^ This review includes the most recently published evidence. Differences in inclusion criteria and in the selection process, when data of multiple studies overlapped, also produce slight variability in studies included.

In comparison with the previous reviews, this reviews focuses on neurologically intact survival, which reflects survival with preserved neurologic function – and quality of life. It also reports outcomes (unadjusted and adjusted) of the individual studies in order to provide transparency. Detailed information of the large variability across studies is provided to show variability in outcome in relation to study setting and patient characteristics. To provide a clear overview, outcomes are illustrated graphically and summary measures are calculated for the different outcome measures.

**References**

1. Eisenberg M, Hallstrom A, Carter W, Cummins R, Bergner L, Pierce J. Emergency CPR instruction via telephone. Am J Public Health. 1985;75:47–50.

2. Kuisma M, Boyd J, Väyrynen T, Repo J, Nousila-Wiik M, Holmström P. Emergency call processing and survival from out-of-hospital ventricular fibrillation. Resuscitation. 2005;67:89–93.

3. Hiltunen P, Silfvast T, Jäntti T, Kuisma M, Kurola J. Emergency dispatch process and patient outcome in bystander-witnessed out-of-hospital cardiac arrest with a shockable rhythm. Eur J Emerg Med. 2015;22:266–72.

4. Goto YY, Maeda T, Goto YY. Impact of dispatcher-assisted bystander cardiopulmonary resuscitation on neurological outcomes in children with out-of-hospital cardiac arrests: a prospective, nationwide, population-based cohort study. J Am Heart Assoc. 2014;3.

5. Oman G, Bury G. Use of telephone CPR advice in Ireland: Uptake by callers and delays in the assessment process. Resuscitation. 2016;102:6–10.

6. Dami F, Carron PN, Praz L, Fuchs V, Yersin B. Why bystanders decline telephone cardiac resuscitation advice. Acad Emerg Med. 2010;17:1012–5.

7. Alfsen D, Møller TP, Egerod I, Lippert FK. Barriers to recognition of out-of-hospital cardiac arrest during emergency medical calls: A qualitative inductive thematic analysis. Scand J Trauma Resusc Emerg Med. 2015;23:1–8.

8. Berdowski J, Beekhuis F, Zwinderman AH, Tijssen JGP, Koster RW. Importance of the First Link Description and Recognition of an Out-of-Hospital Cardiac Arrest in an. Circulation. 2009;119:2096–102.

9. Lewis M, Stubbs BA, Eisenberg MS. Dispatcher-assisted cardiopulmonary resuscitation: Time to identify cardiac arrest and deliver chest compression instructions. Circulation. 2013;128:1522–30.

10. Nikolaou N, Dainty KN, Couper K, Morley P, Tijssen J, Vaillancourt C. A systematic review and meta-analysis of the effect of dispatcher-assisted CPR on outcomes from sudden cardiac arrest in adults and children. Resuscitation. 2019;138:82–105.

11. Bohm K, Vaillancourt C, Charette ML, Dunford J, Castrén M. In patients with out-of-hospital cardiac arrest, does the provision of dispatch cardiopulmonary resuscitation instructions as opposed to no instructions improve outcome: A systematic review of the literature. Resuscitation. 2011;82:1490–5.
